# Identifying airway obstruction in primary care: is there a role for physiotherapists?

**DOI:** 10.1186/s12875-022-01944-z

**Published:** 2022-12-14

**Authors:** Lisa Pagano, Sarah Dennis, Sally Wootton, Sriram Mahadev, Andrew S. L. Chan, Nicholas Zwar, Deborah Pallavicini, Zoe McKeough

**Affiliations:** 1 Sydney School of Health Sciences, Faculty of Medicine and Health, Level 7, D18 - Susan Wakil Health Building, The University of Sydney, Camperdown, NSW 2006 Australia; 2 Ingham Institute for Applied Medical Research, Sydney, Australia; 3 South Western Sydney Local Health District, Liverpool, Australia; 4 Chronic Disease Community Rehabilitation Service, Northern Sydney Local Health District, Sydney, Australia; 5 Royal North Shore Hospital, St Leonards, Australia; 6 Northern Clinical School, University of Sydney, Sydney, Australia; 7 Faculty of Health Sciences and Medicine, Bond University, Gold Coast, Australia; 8 Sydney North Primary Health Network, St Leonards, Australia

**Keywords:** COPD, Primary care, Allied health, Diagnosis, Spirometry

## Abstract

**Aims:**

To examine the implementation of a physiotherapist-driven spirometry case finding service in primary care to identify new cases of COPD and confirm diagnosis of existing cases of COPD.

**Methods:**

Four general practices were recruited. ‘At risk’ participants (aged ≥ 40 years, current/ex-smoker) and people with ‘existing’ COPD were identified from practice databases and invited to attend an assessment with a cardiorespiratory physiotherapist in each general practice. The physiotherapist performed pre/post-bronchodilator spirometry to identify or confirm a diagnosis of COPD (FEV_1_/FVC < 0.7). Outcome measures included number (%) of new cases of COPD, number (%) confirmed diagnosis of COPD and number (%) of high quality spirometry assessments with accurate interpretation.

**Results:**

One hundred forty eight participants (mean age 70 years (SD 11.1), 57% female) attended a baseline assessment (117 ‘at risk’, 31’existing’ COPD) from 748 people invited. Physiotherapists performed 145 pre/post bronchodilator spirometry assessments. Obstruction on post-bronchodilator spirometry was confirmed in 17% (19/114) of ‘at risk’ and 77% (24/31) of ‘existing’ COPD. Majority of cases were classified as GOLD Stage II (63%, *n* = 27). Quality of pre/post bronchodilator spirometries for FEV_1_ were classified as A (68%), B (19%) and C (5%).

**Conclusion:**

Physiotherapists integrated into primary care performed high quality spirometry testing, successfully case finding ‘at risk’ patients and identifying potential misdiagnosis of obstruction in some ‘existing’ COPD cases.

**Trial registration:**

ANZCTR, ACTRN12619001127190. Registered 12 August 2019 – Retrospectively registered, http://www.ANZCTR.org.au/ACTRN12619001127190.aspx

## Background

Primary care is the first point of contact with healthcare systems for most people [1], making it the ideal setting for the early diagnosis and subsequent management of chronic obstructive pulmonary disease (COPD). Yet, there are many challenges in the diagnosis of COPD in primary care with evidence suggesting that there are significant numbers of individuals with undiagnosed airflow obstruction, as well as high levels of misdiagnosis [2–8]. This is often related to variability in the quality of spirometry performed in primary care [9, 10] as well as issues with correct interpretation [11–14]. An additional challenge is that patients can often present later in the disease process when they have become symptomatic [15, 16], further delaying opportunities to intervene. Current guidelines state that spirometry is required to make a diagnosis of COPD with spirometric criterion for airflow obstruction being a post-bronchodilator forced expiratory volume in one second/forced vital capacity (FEV_1_/FVC) fixed ratio of < 0.7 [17, 18]. Despite this, the use of spirometry in primary care has been reported to be suboptimal [7, 12, 19, 20]. The need for high quality and reliable spirometry is imperative to improve detection of COPD as well as classification of severity of disease.

The Global Initiative for Obstructive Lung Disease (GOLD) guidelines advocate for active case finding of COPD in people with symptoms and/or the presence of risk factors [17]. Therefore, increasing the use and uptake of spirometry has been an area of research focus to improve early and accurate diagnosis of COPD. Active case finding for detection of COPD has been shown to be feasible in primary care with identification of new cases of COPD ranging from 1.7% to 30.5% [21]. Many studies have focused on the role of the general practitioner (GP) in screening patients and conducting spirometry [9, 22] or have utilised trained research assistants and trained technicians [23–26]. However, challenges have been reported with these approaches with qualitative evidence reporting that some GPs have perceived difficulties with accurate diagnosis of COPD and lack confidence in the interpretation of spirometry [27–29]. Recent studies have begun to examine the role of other health professionals in undertaking spirometry testing to improve early diagnosis of COPD, such as practice nurses and pharmacists, and these studies have shown promising results in detecting cases of undiagnosed airflow obstruction in primary care [27, 30, 31].

Physiotherapists also contribute to COPD and chronic disease management, such as through the delivery of pulmonary rehabilitation programs and the use and interpretation of spirometry, yet the feasibility of a physiotherapist working in primary care to assist GPs with the diagnosis of COPD has not yet been examined. Considering physiotherapists’ wide skillset in both spirometry and chronic disease management, if successful, there is potential that physiotherapists embedded in primary care could also assist with early intervention where increased service delivery has the potential for substantial health benefit. Therefore, the primary aim of this paper was to examine the implementation of a physiotherapist-driven case finding service in primary care in order to determine if there is a role for physiotherapists in both identifying new cases of COPD and confirming diagnosis and severity in existing cases of COPD. A secondary aim was to examine the feasibility of this service in terms of clinic attendance and the quality of spirometry assessments completed by the physiotherapists with accurate interpretation.

## Methods

A pragmatic cross-sectional study embedded within a larger pilot study was conducted in Sydney, Australia. The study protocol was approved by the Northern Sydney Local Health District Human Research Ethics Committee (HREC reference; HREC/15/HAWKE/434) and was conducted in accordance with the WMA Declaration of Helsinki. The trial was registered with the Australian and New Zealand Clinical Trials Registry (ACTRN12619001127190, registered 12/08/2019). The detailed methods of this study have been published previously [32]. In brief, general practices were invited to participate with assistance from a primary health network. Once written consent was provided by the practice to participate in the study, a senior cardiorespiratory physiotherapist was identified by the local health district to partner with the general practice. The physiotherapists completed a two-hour refresher training workshop in the performance and interpretation of spirometry conducted by members of the study team. People ‘at risk’ of COPD and those with an ‘existing’ COPD diagnosis were considered eligible for inclusion if they met the following criteria: (i) were adults aged 40 years and over; (ii) had attended the practice at least twice with one visit in the preceding 12 months; and (iii) had a documented history of smoking (current or former smoker) in their medical notes or (iv) had a recorded diagnosis of COPD or were taking medications prescribed for COPD (i.e. short acting inhaled β2 agonists (SABA), short acting muscarinic antagonists (SAMA), long acting inhaled β2 agonists (LABA), long acting muscarinic antagonists (LAMA), combination of LABA/LAMA and inhaled corticosteroids). Participants were excluded if they had terminal cancer, a cognitive impairment, required home oxygen, did not speak sufficient English or were pregnant. Potentially eligible participants were identified from a search of the practice electronic records by a research assistant or trained practice staff. The resultant lists were reviewed by the GPs and/or practice nurses who further excluded people on clinical grounds at the practice’s discretion. Examples of reasons for exclusion on clinical grounds included if the staff felt that patients would be unwilling to attend due to life stressors or other medical conditions.

All potentially eligible participants were sent an invitation from the practice inviting them to take part in the study via letter or phone call. After obtaining written informed consent, participants were invited to attend a case finding appointment with the senior cardiorespiratory physiotherapist at the general practice. At the case finding appointment, participants completed baseline demographic questionnaires as well as the COPD Assessment Test (CAT) [33] and Modified Medical Research Council Dyspnoea Scale (mMRC) [34]. All participants then underwent pre and post bronchodilator spirometry using an EasyOne™ diagnostic spirometer (ndd Medical Technologies, Massachusetts, USA or Zurich, Switzerland). Subjects were instructed to withhold all bronchodilators before spirometry. Patient instruction, assessment of acceptability of forced expiratory manoeuvres and criteria for test reproducibility were based on American Thoracic Society/European Respiratory Society (ATS/ERS) guidelines [35]. A minimum of three attempts were required. Best efforts at forced expiration were selected according to the spirometer algorithm and were reviewed by the physiotherapist. Post-bronchodilator spirometry testing was performed 10 to 15 min after 400 mg salbutamol delivered by a metered dose inhaler and spacer. The physiotherapist then determined the presence of obstruction and severity according to GOLD guidelines [17]. A diagnosis of COPD based on GOLD guidelines was assigned to all those participants with a post-bronchodilator FEV_1_/FVC of < 0.7 [17]. Participants in the case finding cohort with obstruction at baseline and those with existing COPD who did not show obstruction were referred back to their GP and encouraged to undergo further testing with a respiratory specialist for confirmation of diagnosis. If spirometry appeared abnormal for other reasons, for example suggesting a restriction defect, the participant was also referred back to their GP.

A member of the study team verified the physiotherapists’ interpretation of the spirometry results according to GOLD classification criteria. Quality of spirometry traces and results were judged according to the ATS/ERS acceptability and repeatability criteria and graded according to the repeatability grading system of quality A to F recommended by the ATS/ERS [36]. The repeatability criteria are applied to the differences between the two largest FVC values and the two largest FEV_1_ values and results are judged separately for pre-bronchodilator and post-bronchodilator results. A ‘grade A’ result constitutes at least 3 acceptable FEV_1_ and FVC manoeuvres with the difference between the two highest readings ≤ 0.150L. Grades B to E vary in number of acceptable manoeuvres and the variability in FEV_1_ and FVC readings. Grade U is classified as useable and grade F is not useable or acceptable [36]. Results that appeared ambiguous or required further interpretation were sent to a respiratory specialist from the research team for review and provision of feedback.

### Outcomes and measurements

The primary outcome measures were the number (%) of new cases of COPD and number (%) of confirmed diagnosis of COPD and severity from previously diagnosed cases. Secondary outcomes consisted of the number (%) of eligible participants invited to attend, the number (%) that attended an appointment with the physiotherapist, the number (%) of spirometry assessments completed by the physiotherapist meeting ATS/ERS criteria [35, 36] with accurate interpretation according to GOLD guidelines and quality of spirometry assessments completed [17]. The ATS recommendations for a standardized pulmonary function report classify grades A, B or C as useable [37]. For this study, grades A, B and C were considered adequate and grades D to F were considered not acceptable for clinical use.

### Statistical analysis

All data are presented as mean ± standard deviation (SD) or number (%) unless otherwise specified for the baseline demographic data and primary and secondary outcomes. Statistical differences between groups at baseline were assessed using chi square tests of homogeneity or Fisher’s Exact tests (categorical variables) and independent t-tests (two-tailed) or Mann–Whitney U tests (continuous or ordinal variables). *P* values < 0.05 were considered statistically significant. Data was analysed using IBM SPSS Statistics for Windows, version 27.0. (IBM Corp., Armonk, N.Y., USA).

## Results

**Fig. 1:**
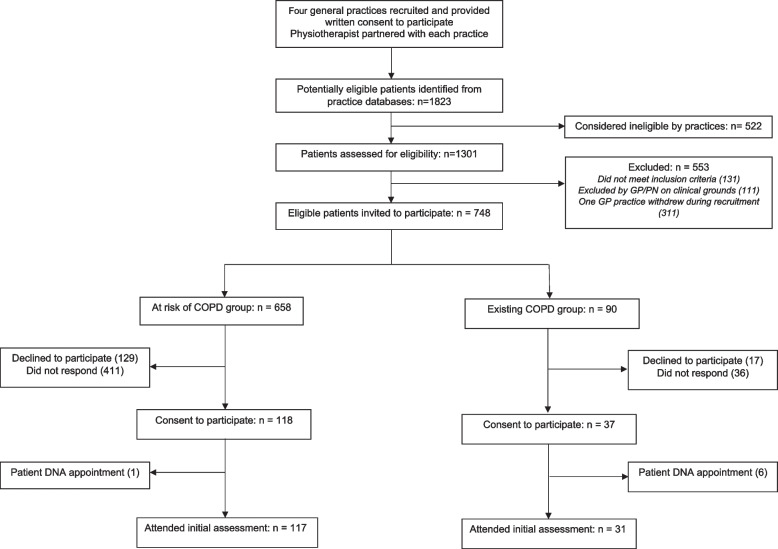
Study Enrolment. Abbreviations: COPD: Chronic Obstructive Pulmonary Disease; DNA: did not attend; GP: general practitioner; PN: practice nurse

A total of four GP practices were recruited and consented to participate in this study (see Fig. 1). Participant recruitment occurred from October 2018 to January 2020. The electronic medical record search in the four practices identified 1823 potentially eligible participants. Of these, 748 participants (*n* = 658 ‘at risk’ of COPD and *n* = 90 with an ‘existing’ COPD diagnosis) were invited to participate in the study. Of those invited, 40% (301/748) responded to the invitation and 21% (155/748) provided written informed consent to participate. A total of 148 (20%, 148/748) participants attended a baseline appointment with the physiotherapist. The physiotherapist completed 145 pre and post bronchodilator spirometry assessments. Three participants were unable to perform spirometry due to poor technique from lack of understanding or the inability to perform despite multiple attempts and instruction. Of the spirometries completed, 114 (79%, 114/145) participants were ‘at risk’ of COPD and 31 (21%, 31/145) had an ‘existing’ COPD diagnosis. The physiotherapist correctly interpreted the level of obstruction according to GOLD classification criteria [17] in 98.6% (143/145) of cases.

The baseline characteristics of the participants are presented in Table 1. The mean age for the total cohort was 70 years (SD 11.1) and 57% (*n* = 84) of the cohort were female. Participants in the ‘existing’ COPD group were significantly older (mean difference (95% CI) 7.9 years (3.7 to 12.2), *p* < 0.001) and had a significantly higher number of comorbidities (*p* < 0.001). The ‘existing’ COPD cohort also reported significantly higher CAT scores at baseline than the ‘at risk’ group (mean difference (95%CI) 6.9 (3.8 to 10.0), *p* < 0.001) and there was a statistically significant difference in mMRC scores between the ‘existing’ COPD group and ‘at risk’ group at baseline (*p* < 0.001).

**Table 1:** Population Characteristics of Subjects

	TOTAL *n* = 148	AT RISK of COPD *n* = 117	EXISTING COPD *n* = 31	*P* value
Mean age, years (SD)	70 (11.1)	68 (11.2)	76 (8.5)	< .001
Gender (% female)	84 (57%)	59 (50%)	25 (81%)	.003
Mean body mass index, Kg/m^2^ (SD)	27.7 (5.3)	27.9 (4.9)	27.2 (6.6)	.474
Median number of co-morbidities [IQR]	4 [2–5]	3 [2–5]	5 [4–9]	< .001
Identify as Aboriginal and/or Torres Strait Islander	4 (3%)	1 (1%)	3 (10%)	.029
English spoken at home	140 (97%)	110 (94%)	30 (97%)	1.00
Currently married	80 (54%)	71 (61%)	9 (29%)	.002
Currently employed	55 (37%)	51 (44%)	4 (13%)	.002
Completed tertiary or vocational degree	93 (63%)	77 (66%)	16 (52%)	.146
Current smokers	16 (11%)	10 (9%)	6 (20%)	0.104
Former smokers	125 (85%)	107 (92%)	18 (58%)	< 0.001
Never smoked	7 (5%)	0 (0%)	1 (23%)	N/A
Mean CAT Score (SD)	10.40 (7.0)	8.92 (6.0)	15.77 (7.9)	< .001
Median mMRC score [IQR]	1 [0–1]	0 [0–1]	1 [1, 2]	< .001

Data are presented as Number (%) unless indicated otherwise

Abbreviations: *CAT, COPD* Assessment Test, *COPD* Chronic obstructive pulmonary disease, *IQR* Interquartile range, *mMRC* Modified medical research council dyspnoea score, *N/A* not applicable, *SD* Standard deviation

The results of the spirometry assessments are shown in Table 2. Airflow obstruction was detected in 30% of participants (*n* = 43) of which 19 participants (44%) were part of the ‘at risk’ of COPD cohort. This indicates a case finding rate of 17% (19/114). The majority of participants were classified as GOLD stage II (63%, *n* = 27). Two participants from the ‘at risk’ group with airflow obstruction were referred by their GP for further follow-up testing for confirmation of diagnosis, of which one had airflow obstruction confirmed and one had a differential diagnosis of asthma. Only one participant in the ‘existing’ COPD group with no airflow obstruction on spirometry underwent further testing and was confirmed as a misdiagnosis.

**Table 2:** Spirometry results

	TOTAL *n* = 145	AT RISK of COPD *n* = 114	EXISTING COPD *n* = 31
Mean post-bronchodilator FEV_1_/FVC (SD)	0.74 (0.1)	0.77 (0.1)	0.62 (0.1)
Mean post-bronchodilator FEV_1_ (SD)	2.43 (0.9)	2.63 (1.0)	1.64 (0.7)
Airway obstruction (FEV_1_/FVC ≤ 0.7)	43 (30%)	19 (17%)	24 (77%)
GOLD Stage I	14 (10%)	9 (8%)	5 (16%%)
GOLD Stage II	27 (19%)	10 (9%)	17 (55%)
GOLD Stage III	2 (1%)	0 (0%)	2 (6%)
GOLD Stage IV	0 (0%)	0 (0%)	0 (0%)
No obstruction	99 (68%)	95 (83%)	7 (23%)

Data are presented as Number (%) unless indicated otherwise

Abbreviations: *COPD* Chronic obstructive pulmonary disease, *FEV*_*1*_ Forced expiratory volume in one second, *FVC* Forced vital capacity, *GOLD* Global initiative for chronic obstructive lung disease, *SD* Standard deviation

COPD GOLD staging classification^17^—Stage 1: FEV_1_ ≥ 80%; Stage 2: FEV_1_ 50–79%; Stage 3: FEV_1_ 30–49%; Stage 4: FEV_1_ < 30%

The quality grading of spirometry assessments according to the ATS/ERS quality criteria for interpretation guidelines [36] is shown in Table 3. The majority of the pre-bronchodilator and post-bronchodilator spirometry results were of adequate quality, classified as grades A, B or C.

**Table 3:** Grading of Spirometry Results

ATS/ERS Grade	Pre-bronchodilator		Post-bronchodilator	
	FEV_1_ *n* = 145	FVC *n* = 145	FEV_1_ *n* = 145	FVC *n* = 145
A	97 (67%)	92 (63%)	101 (68%)	94 (64%)
B	29 (20%)	21 (15%)	26 (18%)	29 (20%)
C	5 (3%)	7 (5%)	9 (6%)	6 (4%)
D	1 (1%)	4 (3%)	2 (1%)	5 (3%)
E	10 (7%)	18 (12%)	5 (3%)	8 (6%)
U-Useable	2 (1%)	0 (0%)	1 (1%)	2 (1%)
F	1 (1%)	3 (2%)	1 (1%)	1 (1%)

Data are presented as Number (%)

Abbreviations: *ATS/ERS* American thoracic society/european respiratory society, *FEV*_*1*_ Forced expiratory volume in one second, *FVC* Forced vital capacity. Grading System for FEV_1_ and FVC^36^

## Discussion

The results of this study demonstrate that experienced senior cardiorespiratory physiotherapists embedded in a primary care practice to conduct case finding assessments via spirometry, are effective in identifying new cases of COPD. Our results also show that the senior cardiorespiratory physiotherapists were successful in interpreting lung function results for new cases of COPD as well as identifying potential misdiagnoses of COPD. To our knowledge, this is the first study to utilise physiotherapists working with GPs in primary care to assist with the identification of COPD.

Our study resulted in a case finding rate of 17%, demonstrating that physiotherapists could be a successful option to integrate into the primary care team to conduct case finding for COPD. Previous studies looking at methods to improve early and accurate diagnosis of COPD within the primary care setting have identified undiagnosed cases, yet some have also reported logistical challenges which could present problems when integrating these methods into clinical practice [22, 24, 27, 38–40]. For example, Zwar et al. (2016) found that practice nurses were able to conduct spirometry to a good standard, yet had difficulty correctly interpreting spirometry results [27]. Similar findings have been reported in studies involving GPs suggesting that GPs themselves may have difficulty with interpretation and grading of results, as well as knowing when to request a spirometry [14, 20, 41]. In our study, the majority of spirometry traces (> 83%) were classified as grades A, B or C which is higher than some studies conducted in primary care in Switzerland and the US where approximately 60% of traces were classified as acceptable for patients with COPD [9, 10]. Our results were also similar to reported standards in Australia where, following two days of centralised training, approximately 88% of pre and post bronchodilator traces were classified as grade A, B, or C [42]. In our study, the physiotherapists were also able to correctly interpret the results and level of airway obstruction according to GOLD classification criteria [17] in the majority of cases. A reason for this accuracy could be that all Australian physiotherapy courses require students to study respiratory physiology and pathophysiology, including of respiratory diseases, and to undertake extensive training in cardiorespiratory skills at a university level [43]. This training includes the use and interpretation of spirometry. We also recruited senior respiratory physiotherapists with at least five years of clinical experience to partner with each general practice meaning they had a high level of skills in relation to assessment and management of people with COPD. It is important to consider that using a specialised cardiorespiratory physiotherapist embedded in a primary care clinic from a local health district may not always be feasible in clinical practice and a more practical option may be using less experienced physiotherapists to partner with general practices. It is likely that private practice physiotherapists who have not had recent cardiorespiratory experience would need re-training in COPD and performance of spirometry in order to conduct case finding. In addition, the physiotherapists used in this study had training at a university level in the performance of spirometry which may not be similar across all educational contexts in other countries. Further research is needed to determine the success of other physiotherapists, such as private practice physiotherapists, in case finding for COPD.

Misdiagnosis and overdiagnosis of COPD remain ongoing issues in primary care [2, 5, 20, 44] and this was highlighted in our study with a proportion of our cohort with an existing COPD diagnosis having no airflow obstruction on spirometry (23%, *n* = 7). Of these, one participant underwent further testing and was confirmed as a misdiagnosis. There are many challenges to the diagnosis of COPD in primary care. Low levels of spirometry use is one such problem with a high proportion of patients with a diagnosis of COPD reported to have no spirometry results documented or have been diagnosed on the basis of non-spirometric criteria alone [45–47]. Studies have also reported persistent treatment based on empiric diagnosis without spirometric confirmation which may result in increased unnecessary cost to the health care system [44, 48]. An additional challenge is the fact that some people have been found to shift between a pattern of obstruction and no obstruction on repeat spirometries which has led some authors to question whether diagnosis of COPD based on spirometry testing at one timepoint is sufficient [49]. It is important to acknowledge that this presents additional challenges in primary care as in Australia, spirometry assessments can only be claimed once in a 12 month period on the Medical Benefits Scheme. Despite this, the integration of a clinician into primary care who is confident with spirometry testing and interpretation could assist in the conduct of valid spirometry tests.

Participant response and uptake are important considerations in determining feasibility of screening programs and enabling effective translation into clinical practice. Overall attendance rate in our study was low with only 21% consenting to participate and 20% attending an appointment. However, this rate is similar to other case finding trials for COPD when using similar methods of recruitment [24, 50, 51]. Barriers to participation in screening initiatives have been reported that remain difficult to address. For example, people who are asymptomatic may not feel the need to participate as evidence suggests people do not present to their GP for review until they are symptomatic [15, 16]. Patient specific factors such as age, time constraints or employment may also impact on attendance and are commonly cited as barriers to uptake of other health screening initiatives such as cervical cancer and colorectal cancer [52, 53]. It was not possible in this pilot study to look at barriers and facilitators to participation in attendees and non-attendees.

Screening completed by other health care professionals in alternative settings to primary care may also be a successful option to improve participation. For example, a pharmacy-based case finding service enabled pharmacists to screen for COPD then refer to GPs as appropriate which resulted in 92% of people approached completing the initial screening assessment [30]. It is also possible that direct communication with participants over the telephone compared to mail invitations may result in higher levels of enrolment by providing the clinician with the opportunity to directly discuss the importance of screening and address patient concerns. Interestingly, in our study, when comparing methods of recruitment, there was little difference in the number of people consenting to participate whether participants received a phone call or a mail invitation (19% vs 16% respectively). However, response rate was substantially higher in the participants that received a phone call (48%) compared to mail invitation (25%). Further research on barriers and enablers to participation in case finding is needed in order to optimise the implementation of screening programs for COPD in Australian general practice.

There were some limitations to this study. This was a feasibility study which only recruited a small number of people at risk of COPD from four general practices which limits the generalisability of findings. In addition, the practices were in a relatively affluent area of Sydney, Australia with generally low rates of smoking [54] so results may not be as easily transferrable to other contexts. The general practices recruited may not be representative of all general practices due to recruitment criteria for the practices and expression of interest in research focused on COPD. Participant recruitment was ceased early in 2020 due to the COVID-19 pandemic outbreak and the restrictions on patients attending for face-to-face GP appointments as well as undergoing spirometry testing. In addition, for optimal performance of spirometry and quality control extensive operator training, including follow-up and supplementary training, routine quality checks of all results and ongoing peer review are recommended [55, 56]. As this was a pilot study, the physiotherapists completed a two-hour training workshop on spirometry at the commencement of the study and peer review was only completed on results that required further interpretation. In our results, there were approximately 13% and 9% of pre-bronchodilator and post-bronchodilator spirometry traces respectively from Grade D to F which was slightly higher than anticipated. Whilst ongoing input for optimal quality control would be preferable, this does represent a more pragmatic approach to case finding in primary care as ongoing quality control may not always be possible in clinical practice.

### Clinical implications

Physiotherapists that are experienced and confident in the interpretation of spirometry could be useful in general practice to support GPs and practice nurses to improve the accuracy and interpretation of spirometry and diagnosis of COPD. The acceptability of this approach has not been explored and constitutes an important topic of future research. This will enable a deeper understanding of the effectiveness of this model as well as barriers and facilitators to implementing this model. It is important to establish if case finding through this approach is cost effective and can lead to early intervention and better health outcomes for patients which in turn, can potentially reduce the burden on healthcare systems. In addition, physiotherapists currently play a key role in management of COPD and other chronic diseases through delivery of pulmonary rehabilitation programs and exercise prescription, as well as secretion clearance techniques. Through integration of physiotherapists into the primary care team, it could provide them with the opportunity for discussions with patients surrounding physical activity and complex behaviour change. It remains to be seen if these discussions in a primary care setting can change behaviour and physical activity levels in people with COPD and lead to a change in disease trajectory.

## Conclusion

The results of this study suggest that experienced cardiorespiratory physiotherapists integrated into primary care can successfully perform and interpret spirometry, identify new cases of airflow obstruction, and assist with confirming diagnosis or misdiagnosis of ‘existing’ cases of COPD. Physiotherapists were able to identify a rate of case finding as well as a proportion of cases with an existing COPD diagnosis with no airflow obstruction that was similar to other studies. Further research is needed to determine if this model is cost effective and can lead to improved COPD management through early intervention.

## Data Availability

Data will be stored according to and as required by the ethics committee. The data that support the findings of this study are not publicly available due to ethics requirements and are only available from the authors upon reasonable request with permission of the Northern Sydney Local Health District Human Research Ethics Committee. All correspondence and material requests should be addressed to the corresponding author Associate Professor Zoe McKeough.
